# One Hospital Department with Three Affiliated Medical Schools - Yea or Nay? A Cross-sectional Survey of Students and Tutors

**DOI:** 10.15694/mep.2020.000093.1

**Published:** 2020-05-06

**Authors:** Jill Cheng Sim Lee, Mingyue Li, Limin Kam, Heng Hao Tan, Kok Hian Tan, Rajeswari Kathirvel

**Affiliations:** 1 KK Women's and Children's Hospital

**Keywords:** medical students, education, obstetrics and gynecology

## Abstract

This article was migrated. The article was marked as recommended.

Background

The experience of teaching students from multiple medical schools with separate curriculums and learning ethos within a single healthcare institution is not well-documented in literature.

Objective

We aimed to identify the benefits and challenges of having students from three medical schools train within one department from the clinical tutors’ and medical students’ viewpoint.

Methods

This was a cross-sectional study at KK Women’s and Children’s Hospital (KKH). A survey was conducted on clinical tutors and students from three medical schools for their viewpoints on a 5-point Likert scale.

Results

91 of 100 (91%) students and 51 of 60 (85%) tutors returned the survey. 95.6% of students and 94.1% of tutors agreed that it was important for KKH to be involved in the teaching of all three medical schools. 83.5% of students and 76.5% of tutors believed they would benefit from shared teaching and learning materials. 84.3% of tutors and 83.6% of students agreed that they could be exposed to new teaching methods while 84.7% of students and 72.5% of tutors believed that opportunities to collaborate between schools would arise. However, 25 (49%) tutors and 58 (63.7%) students believed that there may be limited supervision. Students (60.4%) and tutors (56.9%) alike felt that there would be a lack of learning space.

Conclusion

Both students and tutors believed that it was important for medical students for the department in KKH to be involved in the teaching of all three medical schools. Benefits perceived included shared teaching and learning resources, exposure to new teaching methods and opportunity for collaboration across medical schools. Through careful planning of rotations and supportive leadership, hospitals can optimise teaching capabilities allowing students and tutors to benefit from advantages of teaching of students from multiple medical schools.

## Introduction

KK Women’s and Children’s Hospital (KKH) is the leading training center in Obstetrics and Gynecology (OBGYN) in Singapore, with 12,000 births annually. Singapore has three medical schools: Yong Loo Lin School of Medicine, National University of Singapore (YLL-NUS), Duke-NUS, and Lee Kong Chian School of Medicine, Nanyang Technological University (LKC-NTU). Students from all three schools rotate through KKH for clinical OBGYN experience. There are however finite resources within an institution to support medical student teaching.

The unique experience of teaching multiple medical schools with separate curriculums and learning ethos is not well-documented in literature. In our context, YLL-NUS is the largest and oldest medical school in Singapore, established in 1905 with a traditional 5-year undergraduate curriculum consisting mostly of didactic large-group lectures. Duke-NUS, started in 2005 in partnership with Duke University, North Carolina, conducts a 4-year graduate curriculum with significant focus on research training. LKC-NTU started in 2013, runs a 5-year undergraduate program, collaborating with Imperial College, London using predominantly electronic and team-based learning models. Duke-NUS students commence their clinical OBGYN rotation in Year 2 whilst YLL-NUS and LKC-NTU students commence in Year 4.

This study aims to identify the perceived benefits and challenges of having students from three medical schools train within one department from the clinical tutors and medical students’ viewpoint; the needs of both groups and potential issues which may need to be addressed to prevent negative outcomes for students training in OBGYN, KKH.

## Methods

Two surveys, one for medical students and one for tutors, were developed to collect data from medical students from all three schools and clinical tutors from the Division of OBGYN, KKH, about the presence of three medical schools within a single clinical campus during the academic year of July 2016 to June 2017. The surveys collected data on participant demographics and comprised of a series of statements for their viewpoints on a 5-point Likert scale. Free-text feedback was also collected for qualitative analysis. Surveys were piloted on medical education administrators to ensure ease of use. Paper surveys were distributed to tutors during the department meetings prior to the arrival of students from LKC-NTU, and students received the surveys on the first day of their OBGYN posting.

SingHealth Centralized Institutional Review Board approved this study with an exempt status. Quantitative data were analyzed using Microsoft Excel while qualitative data were analyzed line by line, identifying common keywords and themes using principles of grounded theory.

## Results/Analysis

A total of 91 of 100 (91%) students returned the survey; 28 (30.7%) from YLL-NUS, 11 (12.1%) from Duke-NUS and fifty-two (57.1%) from LKC-NTU while 51 of 60 (85%) clinical tutors returned the survey. Tutor participants consisted of 22 consultants and 29 residents who were tutors for medical students from all 3 medical schools. A third (33.3%) of tutors who responded taught all 3 medical schools, while 39.2%, 15.7%, and 11.8% taught 2, 1 or 0 schools respectively. A total of 87 (95.6%) students and 48 of the sixty tutors surveyed (94.1%) believed it was important for KKH to teach all three medical schools.

**Figure 1.**
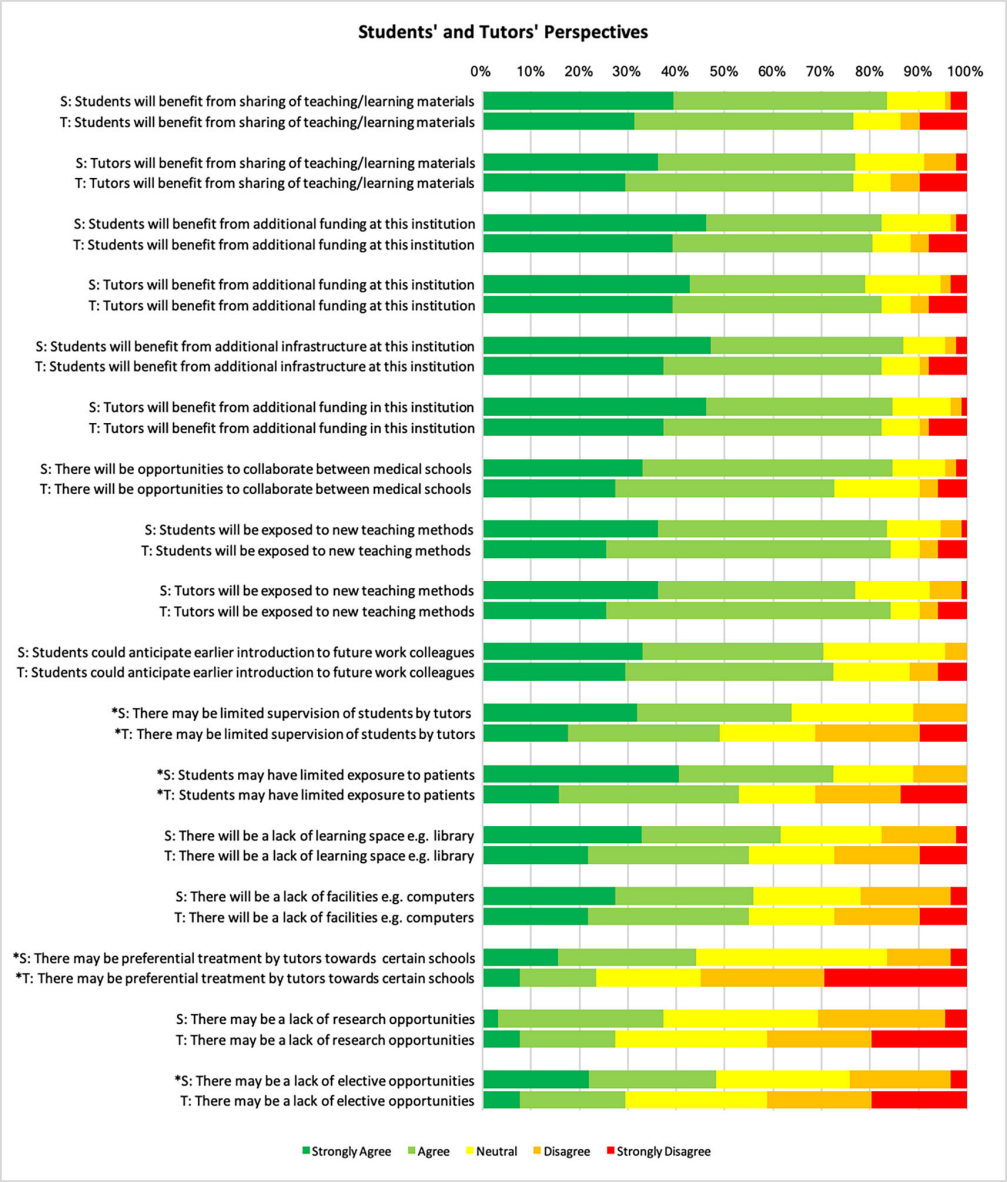
Compares the survey findings between students and tutors.

A total of 76 (83.5%) students and 39 (76.5%) tutors agreed or strongly agreed that both groups would benefit from sharing of teaching and learning materials. 77 (84.7%) students and 37 (72.5%) tutors agreed or strongly agreed that there would be opportunities to collaborate between medical schools (p-value 0.03). 43 (84.3%) tutors and 76 (83.5%) students agreed/strongly agreed that by teaching all 3 medical schools, they could be exposed to new teaching methods. However, 25 (49%) tutors and 58 (63.7%) students believed that there may be limited supervision (p-value 0.03). Students (60.4%) and tutors (56.9%) alike felt that there would be a lack of learning space. Students were more likely than tutors to perceive a lack of patient exposure and elective opportunities (75.8% vs 52.9%, p-value <0.05 and 48.3% vs. 29.4%, p-value <0.05 respectively), whereas the difference in perception of research opportunities was not statistically significant (34.3% vs 27.4%, p-value 0.25). Of the sixty tutors surveyed, 31.4% of tutors thought that they would struggle adjusting between the schools. 44.2% of students and 23.5% of tutors agreed that there may be preferential treatment towards students from certain schools (p-value <0.5)

**Figure 2.**
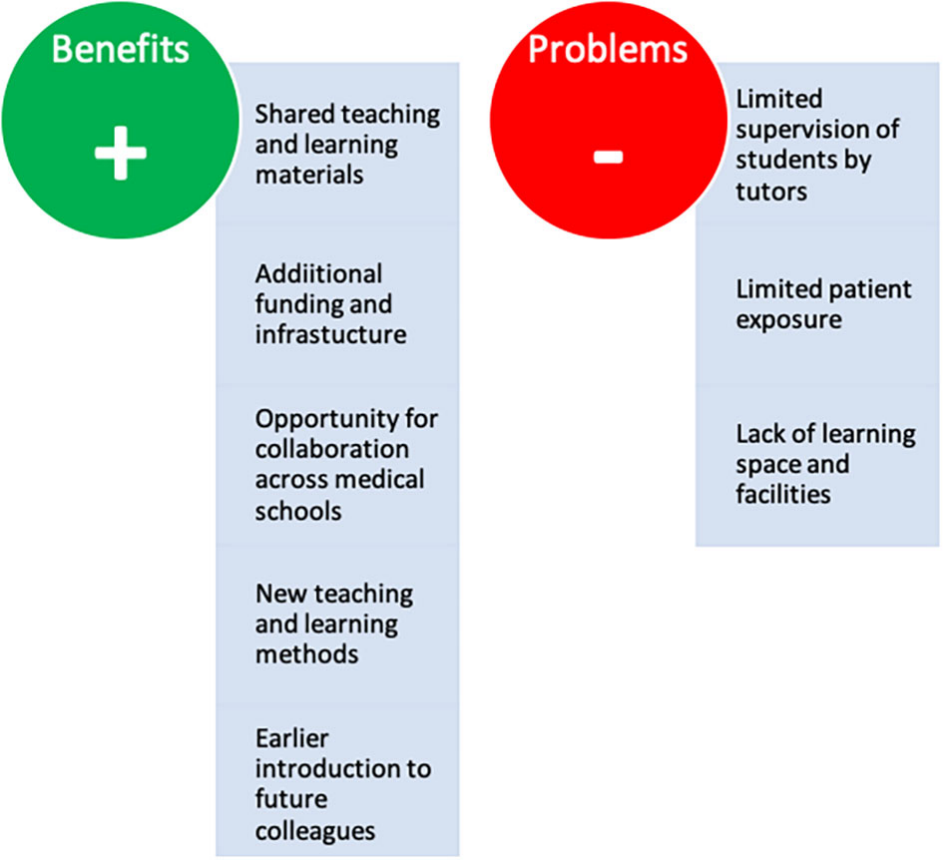
Summarizes the perceived benefits and problems of having a single hospital teaching three medical schools.

Qualitative data analysis revealed that participants agreed that KKH provided ample learning opportunities due to a high patient load and exposure to a wide variety of cases.

**Figure 3.**
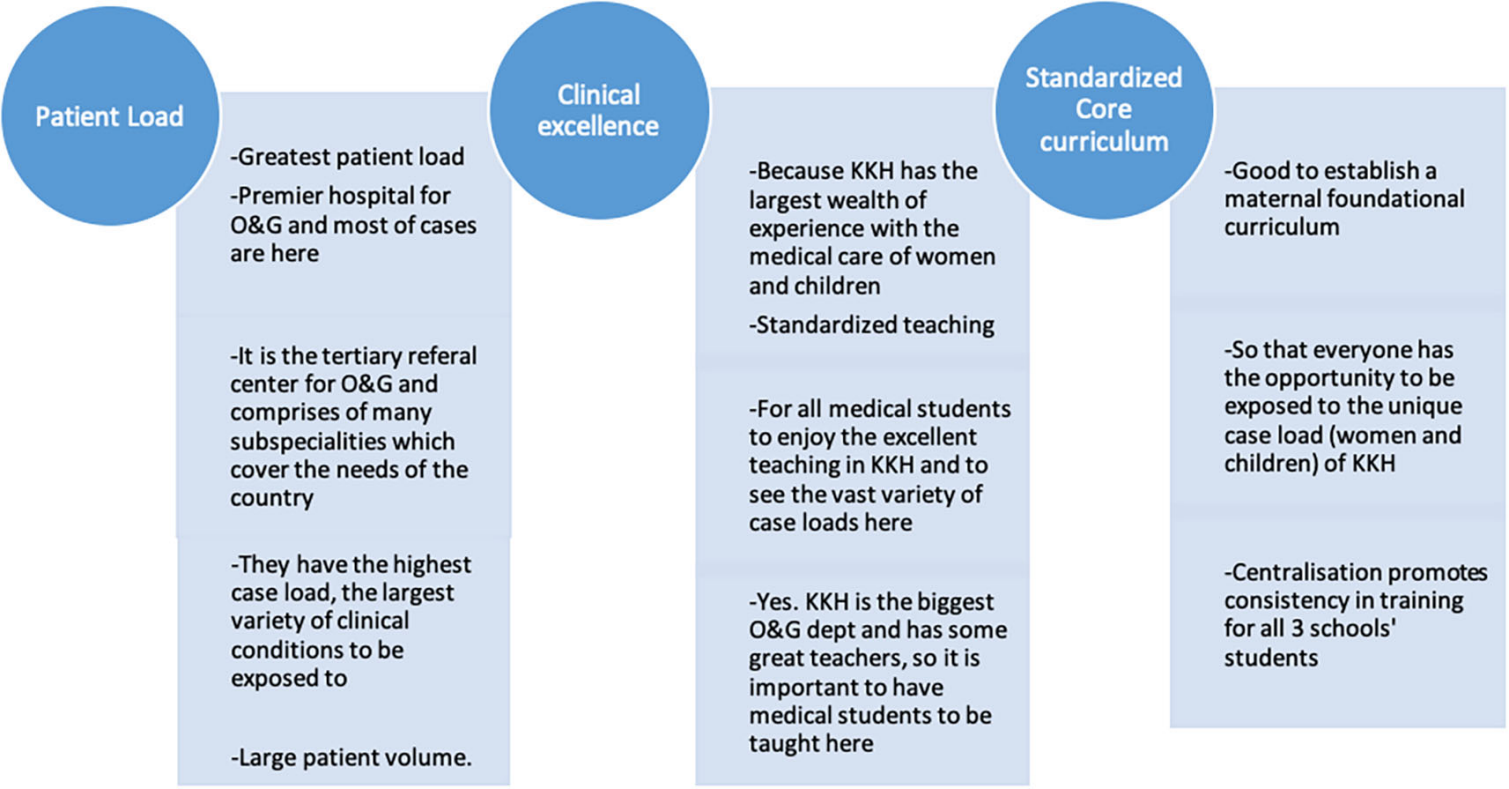
Shows some of the frequent comments that participants made.

## Discussion

Public healthcare institutions carry the responsibility of training medical students, house officers and residents. With increasing life expectancy and healthcare needs of a nation, there is a recognized shortage of doctors ( Grover and Niecko-Najjum, 2013). To meet these demands in Singapore, more medical schools have been set up and more medical students enrolled ( Khalik, 2015). YLL-NUS, Duke-NUS and LKC-NTU enrolled 300 ( National University of Singapore, 2001), 63 ( Duke-NUS Medical School, 2017), and 120 (Lee Kong Chian School of Medicine, 2017) students respectively in 2017. This is a 48.8% rise in intake of medical students at Singapore’s medical schools since 2011 ( Tan, 2017).

Tutors, who train these students, consist of the same pool of doctors who also make up the healthcare staffing deficit. In this study, 11.8% of clinicians did not teach medical students yet recognized the important of leading healthcare institutions participating in medical student teaching. This begs the question, should all public healthcare clinicians teach medical students or should this responsibility be tasked to those with interest of do it better? A 2005 studyfound that clinicians who had not taught, lacked pleasure in teaching and were less likely to have been inspired by powerful mentors during their own training ( Dahlstrom *et al.,* 2005), supporting the premise that clinical teaching should be left to those with interest.

Students and tutors shared similar view on the likely benefits and challenges that were likely to come with teaching three medical schools. The perception mismatch between tutors and students about patient exposure, research and elective opportunities may be explained by the students’ lack of familiarity with the clinical load at our institution.

Sufficient exposure to a specialty is important for medical students to develop interest and future career in that specialty ( Meiboom *et al.*, 2015).

As LKC-NTU and Duke-NUS are relatively young medical schools compared to NUS, students may worry that tutors may have preferential treatment towards other schools. However, tutors do not share similar sentiments and the majority of tutors feel that they will not show preferential treatment.

Training of residents as teachers have repeatedly been shown to be effective in several studies ( Ryg, Hafler and Forster, 2016, Watkins *et al*., 2017, Chokshi *et al*., 2017). It is essential that the medical profession continues to instill the altruism that keeps clinicians training the future generations of doctors. Residents as near-peer tutors provide a good solution to bridge the gap in teaching manpower for large cohorts of students. Our local experience of residents as near-peer mentors has been well-received and highlighted as a positive experience by medical students in their course feedback to the Ministry of Health.

To address the anticipated problems raised from this study, KKH OBGYN has taken the following measures:

• Formal training and updates for clinicians by each school to ensure tutors are well-prepared as educators and remain motivated to teach.
• Extended the role of residents as near-peer tutors to jointly mentor students with senior faculty, conduct small group tutorials and supervise clinic attachments on a one-to-one basis.
• Flipped classroom approach with pre-prepared voice over PowerPoint sides and case-based discussions.
• Co-ordinate rotation calendars across the three schools to reduce overlap of students to no more than two schools at a time.
• Encourage students from multiple schools to attend tutorials conducted by clinician tutors together.
• Secured protected time and teaching awards by the Division to support teaching activities and recognize tutors’ contributions.

In anticipation of rising student numbers, the above measures have helped our institution manage the teaching burden whilst maintaining a good standard of training with excellent student feedback and tutor satisfaction at the end of posting feedback surveys.

The main limitations to this study include that the surveys were not validated prior to administration due to the lack of existing data with regards to similar clinical contexts. The sample size surveyed was also limited by the number of students who have undergone their Obstetrics and Gynecology rotation at KK Women’s and Children’s Hospital in the company of students from the other medical schools as this study was conducted at the time of the introduction of the third medical school.

## Conclusion

Both students and clinical tutors believe it is important to undertake clinical rotations in busy tertiary centers and recognize the benefits and potential concerns with regards to teaching all three medical schools within one center. The differences in perception between students and tutors may be attributed to the lack of familiarity of students with the patient caseloads and elective opportunities at our center.

## Take Home Messages

Concerns about gaps in supervision of medical students may be filled through involvement of residents as near-peer tutors and mentors to medical students. Careful planning of rotations and supportive departmental leaders within such centers can maximize teaching capabilities allowing both students and tutors to benefit from collaborations with multiple medical schools.

## Notes On Contributors

**Jill Cheng Sim LEE**; MBChB, MSc (Clinical Education), MRCOG, MRCP. Associate Consultant, Department of Urogynaecology, Division of Obstetrics and Gynecology, KK Women’s and Children’s Hospital, SingHealth Duke-National University of Singapore (NUS) Obstetrics and Gynecology (OBGYN) Academic Clinical Program, Singapore. Clinical Tutor, Lee Kong Chian School of Medicine, Nanyang Technological University (NTU), Singapore.

**Mingyue LI**; MBBS. Resident, Division of Obstetrics and Gynecology, KK Women’s and Children’s Hospital, SingHealth Duke-NUS OBGYN Academic Clinical Program, Singapore. Core Resident Tutor, Lee Kong Chian School of Medicine, NTU, Singapore.

**Limin KAM**; BComm. Senior Executive, SingHealth Duke-NUS OBGYN Academic Clinical Program, Singapore.

**Heng Hao TAN**; MBBS, MMed (O&G), MRCOG, FAMS. Deputy Chairman of Division of Obstetrics and Gynecology and Head and Senior Consultant of Department of Reproductive Medicine, KK Women’s and Children’s Hospital, SingHealth Duke- NUS OBGYN Academic Clinical Program, Singapore. Adjunct Associate Professor, Lee Kong Chian School of Medicine, NTU, Singapore. Adjunct Associate Professor, Duke-NUS Graduate Medical School and Yong Loo Lin School of Medicine, National University of Singapore, Singapore.

**Kok Hian TAN**; MBBS, MMed (O&G), FRCOG, FAMS. Head of Perinatal Audit and Epidemiology and Senior Consultant of Department of Maternal Fetal Medicine, KK Women’s and Children’s Hospital, SingHealth Duke-NUS OBGYN Academic Clinical Program, Singapore. Group Director, Academic Medicine, SingHealth, Singapore. Senior Associate Dean, Academic Medicine, Duke−NUS Graduate Medical School, Singapore.

**Rajeswari KATHIRVEL**; MBBS, FRCOG. Consultant, Division of Obstetrics and Gynecology, KK Women’s and Children’s Hospital, SingHealth Duke-NUS OBGYN Academic Clinical Program, Singapore. Adjunct Assistant Professor, Lee Kong Chian School of Medicine, NTU, Singapore. Adjunct Assistant Professor, Duke-NUS and Yong Loo Lin School of Medicine, National University of Singapore, Singapore.

## References

[ref1] Chokshi B. D. Schumacher H. K. Reese K. Bhansali P. (2017) A “Resident-as-Teacher” Curriculum Using a Flipped Classroom Approach: Can a Model Designed for Efficiency Also Be Effective? Academic Medicine. 92(4), pp.511–514. 10.1097/ACM.0000000000001534 28030417

[ref2] Dahlstrom J. Dorai-Raj A. McGill D. Owen C. (2005). What motivates senior clinicians to teach medical students? BMC Medical Education. 5(1), pp.27. 10.1186/1472-6920-5-27 16022738 PMC1185542

[ref3] Duke-NUS Medical School . (2017) Class Profile. Available at: https://www.duke-nus.edu.sg/admissions/student-information/class-profile( Accessed: 10 November 2018).

[ref4] Grover A. and Niecko-Najjum L. M. (2013) Building A Health Care Workforce For The Future: More Physicians, Professional Reforms, And Technological Advances. Health Affairs. 32(11), pp.1922–1927. 10.1377/hlthaff.2013.0557 24191081

[ref5] Khalik S. (2015) Number of foreign doctors rising in Singapore public hospitals and polyclinics. Available at: https://www.straitstimes.com/singapore/health/number-of-foreign-doctors-rising-in-singapore-public-hospitals-and-polyclinics( Accessed: 07 November 2018).

[ref6] Lee Kong Chian School of Medicine . (2018) FAQs. Available at: http://www.lkcmedicine.ntu.edu.sg/aboutus/Pages/FAQs.aspx( Accessed: 10 November 2018).

[ref7] Meiboom A. A. de Vries H. Hertogh C. M. P. M. and Scheele F. (2015) Why medical students do not choose a career in geriatrics: a systematic review. BMC Medical Education. 15(101). 10.1186/s12909-015-0384-4 PMC4470031 26043772

[ref8] National University of Singapore Yong Loo Lin School of Medicine (2001). FAQs. Available at: http://nusmedicine.nus.edu.sg/admissions/medicine/undergraduate#faq( Accessed: 10 November 2018).

[ref9] Ryg P. A. Hafler J. P. and Forster S. H. (2016) The Efficacy of Residents as Teachers in an Ophthalmology Module. Journal of Surgical Education. 73(2), pp.323–328. 10.1016/j.jsurg.2015.10.014 26774939

[ref10] Tan T. (2017) Medical school places to rise to 500 next year. Available at: https://www.straitstimes.com/singapore/education/med-school-places-to-rise-to-500-by-next-year( Accessed: 10 November 2018).

[ref11] Watkins A. A. Gondek S. P. Lagisetty K. H. Castillo-Angeles M. (2017) Weekly e-mailed teaching tips and reading material influence teaching among general surgery residents. The American Journal of Surgery. 213(1), pp.195–201. 10.1016/j.amjsurg.2016.05.004 27640910

