# Supplementary material for: One Hospital Department with Three Affiliated Medical Schools - Yea or Nay? A Cross-sectional Survey of Students and Tutors
Source: MedEdPublish (2016). 2020 May 6;9:93. [Version 1] doi: 10.15694/mep.2020.000093.1 (PMC10697455; doi:10.15694/mep.2020.000093.1)
Supplement: Peer review history

# Reviewer report: Reviewer response for version 1

**DOI:** 10.21956/mep.20020.r30550

**Published:** 2020-05-19

**Authors:** Mohamed Al-Eraky

**Affiliations:** 1 University of Dammam

This review has been migrated. The reviewer awarded 3 stars out of 5 It’s not uncommon to accommodate students from different medical schools to be trained at one teaching hospital. The article reported satisfaction of students and tutors. I would suggest to investigate another aspect within the same setting. The real challenge is to tailor field training to meet the expectations of the academic program and achieve the learning outcomes of three medical schools, together. I wonder if this is possible. Alliteratively, hospital administration may simply decide to impose their training agenda on the three medical schools. These is now single best answer to this dilemma. The question is: To what extent, academic faculty members and hospital tutors are willing to compromise to come up with a common agenda for training of medical students. Thank you and good luck.

Reviewer Expertise:

NA

No decision status is available

# Reviewer report: Reviewer response for version 1

**DOI:** 10.21956/mep.20020.r30548

**Published:** 2020-05-11

**Authors:** James Gray

**Affiliations:** 1 University of Sheffield

This review has been migrated. The reviewer awarded 3 stars out of 5 This is an interesting paper in an area that is seen in parts of the UK with various Medical Schools "sharing" trusts. The study gives interesting findings although I felt that some more detail of the qualitative analysis may have added additional richness to the results and the message as a whole as I felt that the quantitative results rather dominated here. I fully support the comments about residents as tutors - there is good evidence for the community of practice element of newer doctors supporting students and the benefits this holds but the section in discussion did not seem to naturally fit with the results and it may benefit from a rethink as how this relates to the work as a whole.There are some minor language issues that would benefit from a review but overall an interesting article.

Reviewer Expertise:

NA

No decision status is available

# Reviewer report: Reviewer response for version 1

**DOI:** 10.21956/mep.20020.r30549

**Published:** 2020-05-08

**Authors:** P Ravi Shankar

**Affiliations:** 1 American International Medical University

This review has been migrated. The reviewer awarded 4 stars out of 5 I found this article to be of interest. I have worked for a number of years at offshore Caribbean medical schools which usually do not own hospitals. Students from these schools do their clinical rotations at hospitals in the United States and in Jamaica and Barbados in the Caribbean. Some schools are also exploring the possibility of doing clinical rotations in Cuba. These hospitals have students from a number of medical schools and the challenges mentioned by the authors are manifested. The accrediting agencies recommend each school have a dedicated group of faculty and tutors within each clinical department and regular interactions between the school’s academic leadership and the clinical faculty. They also recommend that these faculty be appointed as part-time clinical faculty by the school. The paper is well-written though there are language errors in two or three places which can be corrected. The diagrams illustrate well important concepts discussed in the text while figure 3 illustrates the comments by the respondents. I enjoyed reading this paper and faculty of schools who share clinical teaching spaces with other schools will find this article to be of special interest.

Reviewer Expertise:

NA

No decision status is available
